# Hip region muscular dystrophy and emergence of motor deficits in dysferlin‐deficient Bla/J mice

**DOI:** 10.14814/phy2.13173

**Published:** 2017-03-21

**Authors:** Nadia Nagy, Randal J. Nonneman, Telmo Llanga, Catherine F. Dial, Natallia V. Riddick, Tom Hampton, Sheryl S. Moy, Kimmo K. Lehtimäki, Toni Ahtoniemi, Jukka Puoliväli, Hillarie Windish, Douglas Albrecht, Isabelle Richard, Matthew L. Hirsch

**Affiliations:** ^1^ Gene Therapy CenterUniversity of North Carolina at Chapel HillChapel HillNorth Carolina; ^2^ Department of OphthalmologyUniversity of North CarolinaChapel HillNorth Carolina; ^3^ Department of PsychiatryUniversity of North CarolinaChapel HillNorth Carolina; ^4^ Mouse SpecificsBostonMassachusetts; ^5^ Charles River DiscoveryKuopioFinland; ^6^ Jain Foundation, Inc.SeattleWashington; ^7^ Généthon [IR1] INSERMU951INTEGRARE Research UnitEvryFrance

**Keywords:** Bla/J, dysferlin, locomotor deficits, mouse model, muscular MR imaging and spectroscopy

## Abstract

The identification of a dysferlin‐deficient animal model that accurately displays both the physiological and behavior aspects of human dysferlinopathy is critical for the evaluation of potential therapeutics. Disease progression in dysferlin‐deficient mice is relatively mild, compared to the debilitating human disease which manifests in impairment of particular motor functions. Since there are no other known models of dysferlinopathy in other species, locomotor proficiency and muscular anatomy through MRI (both lower leg and hip region) were evaluated in dysferlin‐deficient B6.A‐*Dysf* ^*prmd*^/GeneJ (Bla/J) mice to define disease parameters for therapeutic assessment. Despite the early and progressive gluteal muscle dystrophy and significant fatty acid accumulation, the emergence of significant motor function deficits was apparent at approximately 1 year of age for standard motor challenges including the rotarod, a marble bury test, grip strength, and swimming speed. Earlier observations of decreased performance for Bla/J mice were evident during extended monitoring of overall exploration and rearing activity. Comprehensive treadmill gait analyses of the Bla/J model indicated significant differences in paw placement angles and stance in relation to speed and platform slope. At 18 months of age, there was no significant difference in the life expectancy of Bla/J mice compared to wild type. Consistent with progressive volume loss and fatty acid accumulation in the hip region observed by MRI, mass measurement of individual muscles confirmed gluteal and psoas muscles were the only muscles demonstrating a significant decrease in muscle mass, which is analogous to hip‐girdle weakness observed in human dysferlin‐deficient patients. Collectively, this longitudinal analysis identifies consistent disease parameters that can be indicators of efficacy in studies developing treatments for human dysferlin deficiency.

## Introduction

Muscular dystrophies are a group of progressive muscular diseases characterized by muscular atrophy, each with a distinct pattern of muscle weakness, onset, and severity. Dysferlinopathies, including limb‐girdle muscular dystrophy type 2B (LGMD2B) and Miyoshi Myopathy, result from a loss of dysferlin, a membrane‐associated protein shown to mediate calcium‐dependent sarcolemma repair, vesicle trafficking, and cell adhesion (Bansal et al. 2003; Glover and Brown 2007; Morree et al. 2013). The absence of dysferlin‐mediated repair results in progressive muscular degeneration due to necrosis of muscle fibers (Bansal et al. 2003). Dysferlin‐deficient patients often present with elevated creatine kinase in circulation and experience slow progression of muscular weakness curiously beginning in the late second decade of life, with most patients requiring a wheelchair for mobility by the age of 40 (Aoki 1993). There is no known cure for dysferlinopathy and its incidence remains poorly characterized yet more prevalent than originally thought as increasingly diagnosed by genetic testing. Clinical management of dysferlinopathy is currently limited to physical, occupational, and psychological therapy to reduce complications of immobility and promote productive, functional patient independence.

While there is no cure for dysferlinopathy, there are several therapeutic avenues under investigation for which relevant representative animal models, showing patient‐like pathologies, are necessary to determine efficacy (Krahn et al. 2009; Lostal et al. 2010; Wein et al. 2010; Grose et al. 2012; Hirsch et al. 2013; Dominov et al. 2014; Pryadkina et al. 2015; Sondergaard et al. 2015). Two naturally occurring mouse models of dysferlinopathy have been widely used: the A/J and SJL/J. The A/J model results from a retrotransposon insertion in intron 4 resulting in a complete loss of dysferlin, while the SJL/J model has a natural splice site mutation in exon 45 resulting in 85% reduction in full‐length dysferlin (the deletion removes the C2E domain) (Bittner et al. 1999). Despite the complete absence of dysferlin, the A/J model shows a later onset of abnormalities in muscle histology and a milder phenotype than SJL/J mice (Ho et al. 2004). As these models are naturally occurring, the appropriate genetic control mice were difficult to define. In a search for better dysferlinopathy models, transgenic mice were developed. These knockouts include the Dysf^−/−^, 129‐Dysf^tm1Kcam^/J, which show similar pathologies and disease progression as the SJL/J mice (Ho et al. 2004), but which present a genotyping challenge that was only recently resolved (Wiktorowicz et al. 2015).

To refine a dysferlin‐deficient animal model, the B6.A‐Dysf^prmd^/GeneJ (Bla/J) mouse was developed by backcrossing the A/J model onto the C57BL/6 strain (Lostal et al. 2010). This allowed a wild‐type parental strain with the same genetic background to serve as a control. The Bla/J model is reported to show similar pathological features as the A/J model; however, Bla/J mice demonstrate significantly more gastrocnemius central nucleation, a marker of muscle regeneration consequently implying turnover (Lostal et al. 2010). This is consistent with the more distal pathology seen in human Miyoshi Myopathy (Lostal et al. 2010; Hornsey et al. 2013). Unlike the A/J mouse model, the Bla/J model presents an advantage, as it is not C5 complement deficient, and in consequence, it is less susceptible to immune disorders and infections than the A/J model (Rhodes et al. 1980; Lostal et al. 2010).

In this study, the disease progression of Bla/J mice was evaluated by standard challenges analyzing locomotor ability, coordination, strength, and magnetic resonance (MR) imaging and spectroscopy. These data demonstrate that although significant deficits could be observed as early as 15 weeks of age in the Bla/J mouse, the majority of disease parameters were evident after 1 year. In addition, comprehensive treadmill gait analyses of the Bla/J model indicated significant differences in paw placement angles and stance in relation to speed and platform slope. MR imaging identified predominantly gluteal and psoas muscle atrophy, with prominent fat infiltration. This study describes the emergence of a progressive hip region muscular dystrophy in Bla/J dysferlin‐deficient mice and relates muscle atrophy to decreased physical abilites.

## Materials and Methods

### Animal models

**Figure 1:**
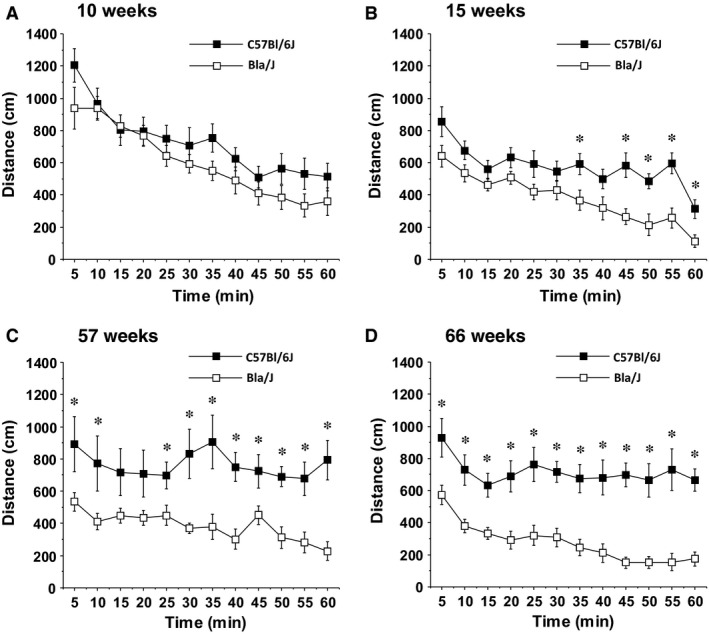
Locomotor deficits in Bla/J mice tested in an open field. Data shown are means ± SEM for each group for a 1‐h test session. *P < 0.05.

**Figure 2:**
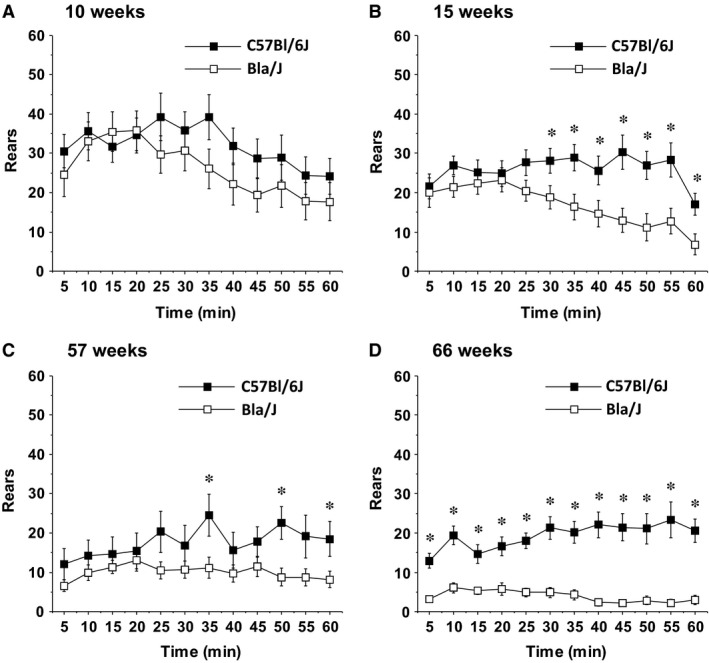
Reduced rearing movements in Bla/J mice tested in an open field. Data shown are means ± SEM for each group for a 1‐h test session. *P < 0.05.

**Figure 3:**
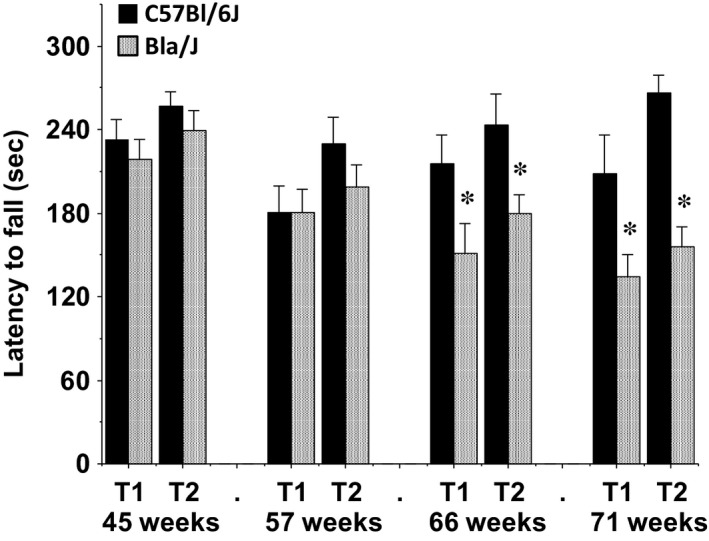
Impaired motor coordination in Bla/J mice tested on an accelerating rotarod. Mice were given two trials (T1 and T2) at each time point. Maximum trial length was 5 min. Data shown are means + SEM. *P < 0.05.

**Figure 4:**
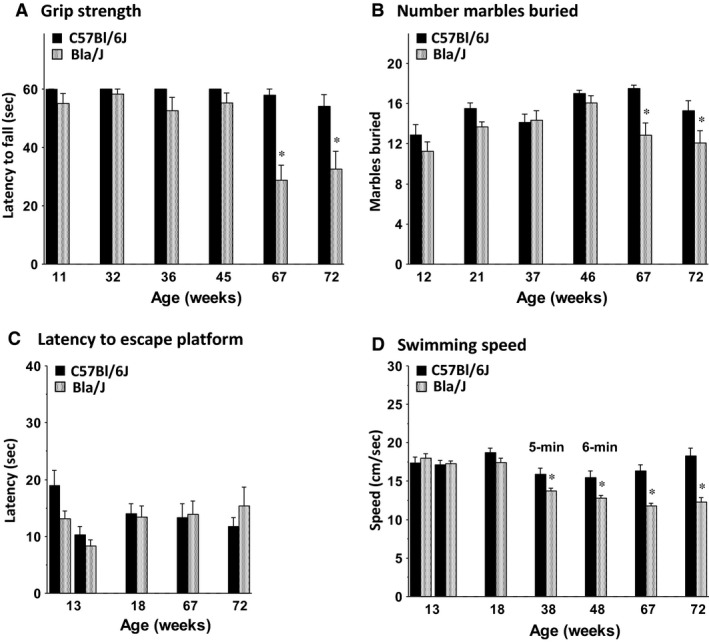
Emergence of motor deficits in Bla/J mice. Data are means ± SEM for (A) grip strength in a 1‐min wire hang task, (B) digging in a 30‐min marble‐burying task, (C) latency to a visible platform in a Morris water maze, and (D) swimming speed during a visible platform task (weeks 13, 18, 67, and 72) and during a 5‐min or 6‐min free swim (weeks 38 and 48). Data for the visible platform task are the mean + SEM) of four 1‐min trials per day. One Bla/J male mouse was not tested in the 6‐min free swim because of a skin lesion. *P < 0.05.

**Figure 5:**
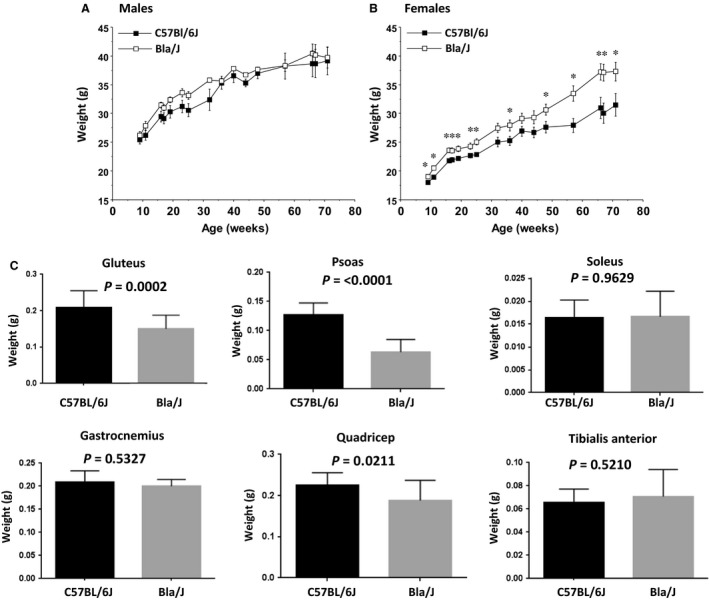
Longitudinal evaluation of total mouse body mass and select wet muscle mass in Bla/J mice. Recorded weekly live mouse body mass for C57BL/6J compared to Bla/J mice separated by gender showed BLA/J males did not differ in body mass from C57BL/6J control mice (A), while BLA/J females (B) body mass was significantly higher than C57BL/6J genetic background control mice over almost all time points, with means increasingly differing past 50 weeks of age. Initial number of mice (weeks 9–72) were six male and eight female C57BL/6J controls and eight male and eight female Bla/J. Data shown are means + SEM for each group. *P < 0.05. (C) Muscle mass for specific muscles were compared in a separate cohort of male mice (*N* = 16) for each genotype. Mice were sacrificed at 12 months of age.

**Figure 6:**
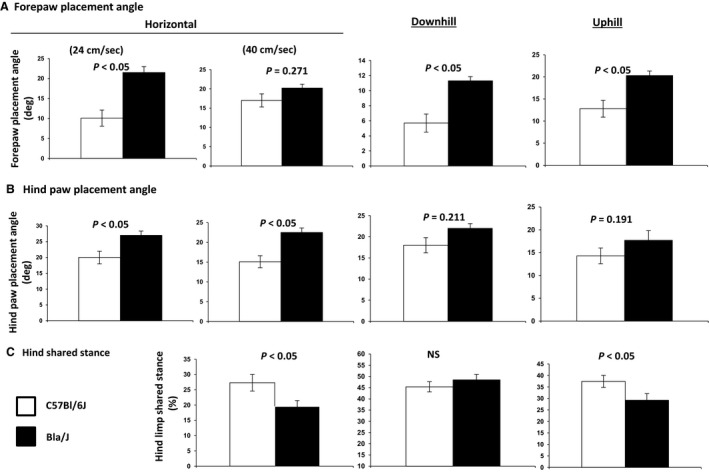
Select gait indices invoking neuromuscular function. Mice were evaluated at 25 weeks. (A) Forepaw placement angle was significantly more open in Bla/J mice compared to WT mice, differences that were sustained during uphill and downhill walking. (B) Hind paw placement angle during full stance was more open during horizontal walking at 24 cm/s and 40 cm/s. Hind paw placement angle was less adaptive in Bla/J mice to changes in walking conditions. (C) Hind limb shared stance was significantly briefer in Bla/J mice than in WT mice, suggesting muscle weakness. This characteristic was more apparent in downhill walking than during uphill walking.

There were four separate mice groups present in this study. The first group of subjects were 14 C57BL/6J mice (6 males and 8 females) and 16 Bla/J mice (8 males and 8 females) on a C57BL/6J background (Jackson Laboratory) for a total of 30 mice, these mice provided data for Figures 1, 2, 3, 4, 5B and 6. The second group of mice were 6 C57bl6J and 6 BLA/J in the experiments of Fig. 6. The third group of subjects were 16 C57bl/6J and 16 BLA/J male mice on a C57BL/6J background, for a total of 32 mice which were used in Figure 5C. The fourth group consisted on 16 mice (8 males, 9 females) for each genotype, for a total of 32 mice. The third group of mice were used for Figures 8, 9, 10, 11, 12. The fourth group of mice (*N* = 6 males/group) generated the data for Figure 6. Subjects were group‐housed in ventilated cages with free access to water and mouse chow. The housing room was maintained on a 12L:12D circadian schedule with lights on at 7 am. All testing procedures were conducted in strict compliance with the Guide for the Care and Use of Laboratory Animals (Institute of Laboratory Animal Resources, National Research Council, 1996) and approved by the Institutional Animal Care and Use Committee of the University of North Carolina.

**Figure 7:**
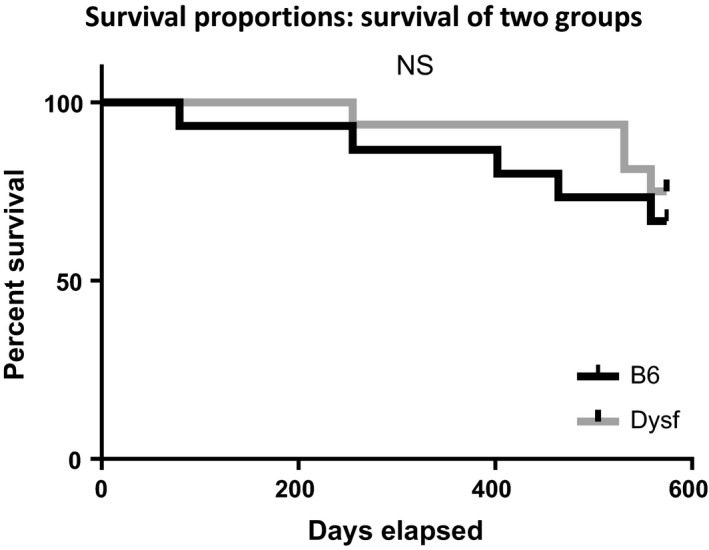
Kaplan–Meier survival curve. There were no significant differences in survival between Bla/J and C57B6 mice up to sacrifice at 82 weeks of age by log‐rank (Mantel–Cox) test analysis (*P* = 0.53) or Grehan–Breslow–Wilcoxon test analysis (*P* = 0.48).

**Figure 8:**
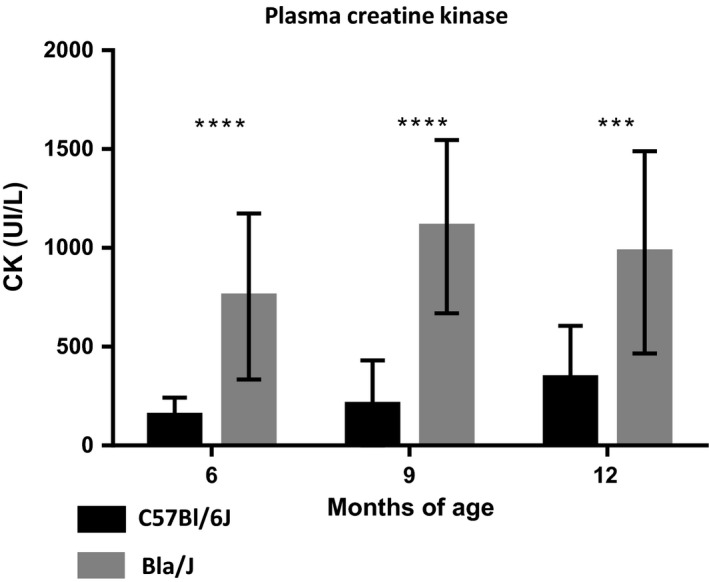
Circulating creatine kinase activity in WT and Bla/J mice. Blood samples were taken at the indicated time points from the indicated mouse models and analyzed for creatine kinase activity (*N* = 16 per genotype and equally divided by gender). Data are presented as mean ± SEM. ***P < 0.001, ****P < 0.0001.

**Figure 9:**
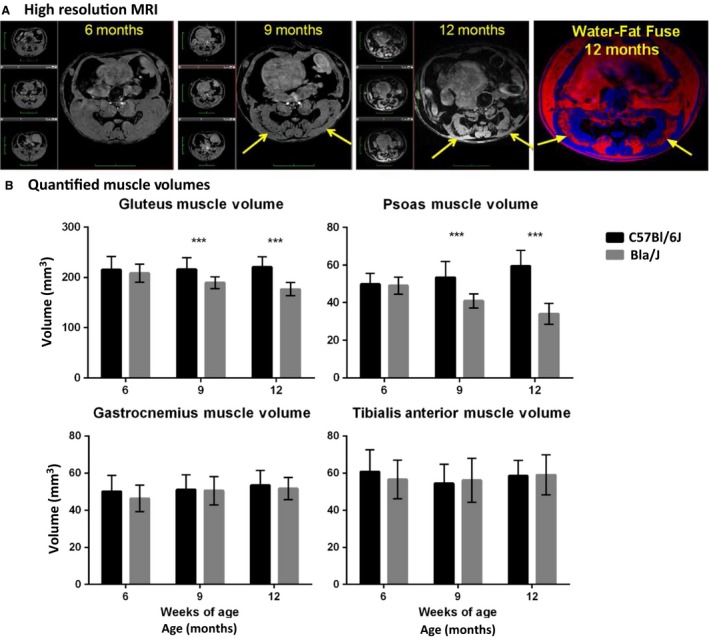
MRI evaluation. (A) Longitudinal high‐resolution MRI images of single Bla/J female mouse over 6–12 months observation period (gray scale images) and fused water (blue color) and fat (red) images. Yellow arrows point to the regions where muscle wasting and fat accumulation occurs. Black bands in gray scale images are assigned as fat, which is confirmed by water/fat MR imaging experiment (same regions seen in fat images, red). (B) Quantified muscle volumes for hip region; gluteus and psoas muscles, and lower leg muscles; gastrocnemius and tibialis anterior. Data (*N* = 16 for both groups) are shown as mean + SEM, unpaired nonparametric Mann–Whitney test comparing ranks, ***P <* *0.0005.

**Figure 10:**
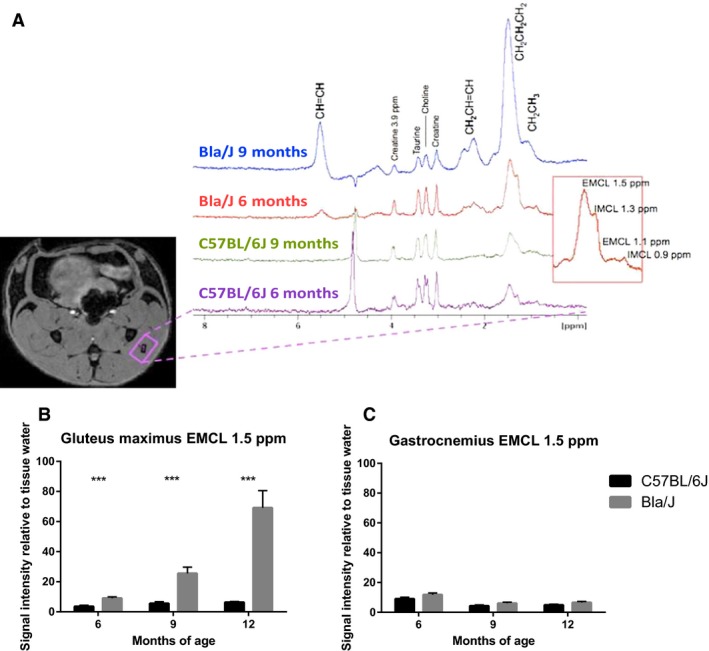
Magnetic resonance spectroscopy of Bla/J muscle. (A) Location of a MR spectroscopic voxel in gluteus maximus muscle and stacked 1H‐MR spectra from 6 and 9 months old C57BL/6J and Bla/J mice showing increased and progressive levels of fatty acids in Bla/J mice (red box; assignment of extramyocellular lipids [EMCL] and intramyocellular lipids [IMCL] lipids, different lipid resonance assigned as per protons in a fatty acid chain). (B) Increase of quantified EMCL 1.5 ppm resonance in the gluteus muscle and (C) quantified EMCL 1.5 ppm resonance in gastrocnemius muscle. Data (*N* = 16 for both groups) are shown as mean + SEM, unpaired nonparametric Mann–Whitney test comparing ranks, ***P <* *0.0005.

**Figure 11:**
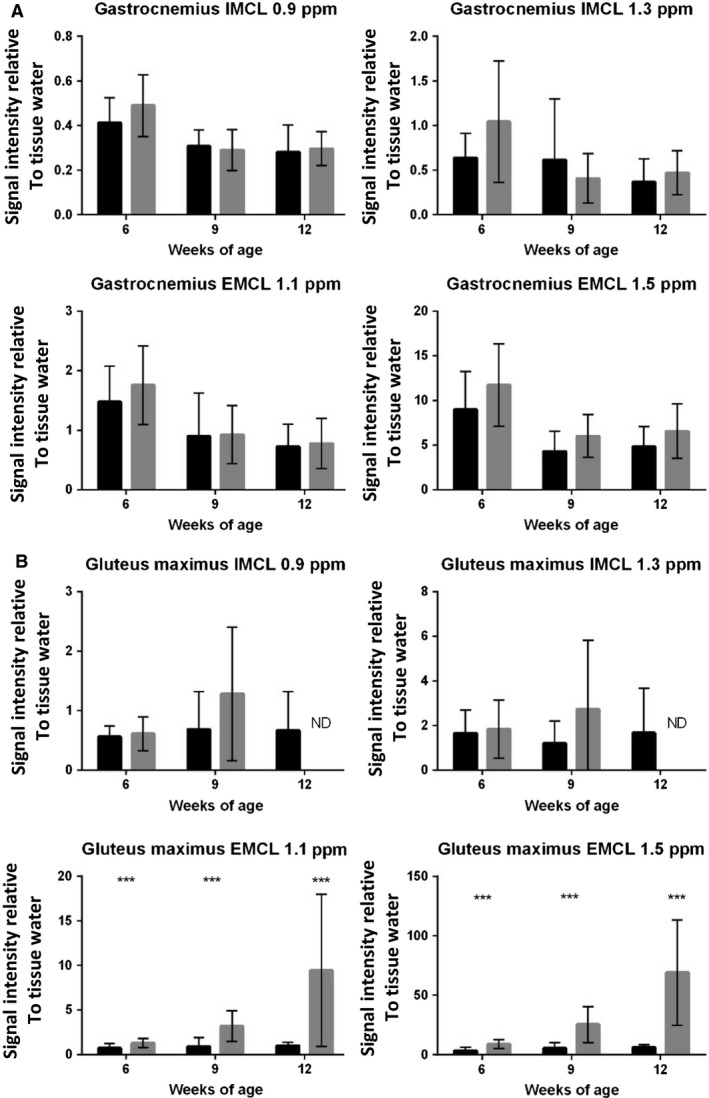
Extramyocellular lipid accumulation in Bla/J muscle over time. (A) Quantified levels of IMCL 0.9 and 1.3 ppm, and EMCL 1.1 and 1.5 ppm IMCL signal intensities in the gastrocnemius muscle. (B) Quantified levels of IMCL 0.9 and 1.3 ppm, and EMCL 1.1 and 1.5 ppm IMCL signal intensities in the gluteus muscle. Data (*N* = 16 for both groups) are shown as mean + SEM, unpaired nonparametric Mann–Whitney test comparing ranks, ***P <* *0.0005.

**Figure 12:**
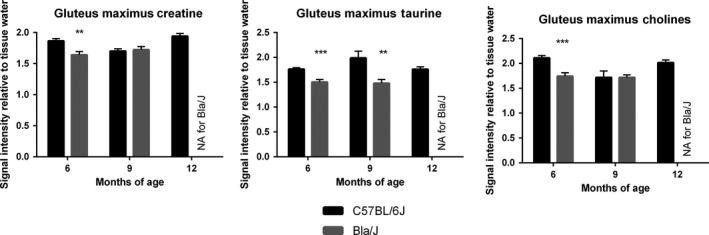
Magnetic resonance analysis of metabolites. (A) Creatine and choline were found to be significantly lower in Bla/J mice at 6 months of age, while taurine was significantly lower in Bla/J mice at 6 and 9 months.

### Behavioral testing procedures

From 10 to 13 weeks of age, mouse activity levels were tested in an open field, motor coordination on an accelerating rotarod, grip strength in a wire hang test, digging in a marble‐burying assay, and vision and swimming ability in a Morris water maze test. Only one procedure was conducted each day. Mice were retested approximately once per month on each assay for the following 60 weeks to detect the emergence of motor deficits or other behavioral changes in the Bla/J group.

**Table 1:** Assay List

	Earliest tested significance	Age (weeks)
Creatine kinase	****	26
Exploration	**	15
Rearing	*	10
Rotarod	**	66
Wire hang test	**	67
Marble burying	*	67
Grip strength	**	67
Swim speed	**	38
Male body mass	ns	10–72
Female body mass	*	10
Psoas wet mass	****	52^
Gluteus wet mass	***	52^
Quadricep wet mass	*	52^
Gastrocnemius WET MASS	ns	52^
Soleus wet mass	ns	52^
Tibialis anterior wet mass	ns	52^
Forepaw angle horizontal 24 m/sec	*	26
Forepaw angle horizontal 40 m/sec	ns	26
Forepaw angle downhill	*	26
Forepaw angle uphill	*	26
Hind paw angle horizontal 24 m/sec	*	26
Hind paw angle horizontal 40 m/sec	*	26
Hind paw angle uphill and downhill	ns	26
Hind shared stance horizontal	*	26
Hind shared stance downhill	ns	26
Hind shared stance uphill	*	26
MRI volume gluteus	***	39
MRI volume psoas	***	39
MRI volume gastrocnemius	ns	26–52
MRI volume tibialis anterior	ns	26–52
MRS gluteus IMCL 0.9 and 1.3	ns	26–52
MRS gluteus EMCL 1.5	***	26
MRS gluteus EMCL 1.1	***	26
Mouse mortality	ns	0–80

List of assays earliest significant evaluated time point, all assays listed were significant at later time points. *P < 0.05, **P < 0.01, ***P < 0.001, ****P < 0.0001. ^^^ denotes terminal point assay; ns, not significant.

### Creatine kinase measurement

Approximately 100 *μ*l of blood was collected in EDTA tubes through the saphenous vein of awake mice and stored in an ice bath. Samples were centrifuged at 2000*g* for 10 min at 4°C. Plasma was collected, frozen immediately on liquid nitrogen, and stored at −80°C. Creatine kinase levels were evaluated in batches using a Konelab T series CK (IFCC) unit. Creatine kinase data were evaluated by an unpaired, nonparametric Mann–Whitney test comparing ranks using Graphpad Prism software.

### High‐resolution magnetic resonance imaging and 1H‐magnetic resonance spectroscopy

Mice (*N* = 16 for both C57BL/6J and Bla/J; 8 females, 8 males) were subjected to MRI and 1H‐MRS at ages 6, 9, and 12 months to assess longitudinal changes in muscle volumes, fat accumulation, and MR spectroscopic changes. The volumes of gluteus maximus and psoas muscles from the hip and gastrocnemius and tibialis anterior from lower leg were determined from anatomical 3D MRI scan followed by 1H‐MRS scan from the gluteus maximus and gastrocnemius (two separate imaging sessions).

MRI/MRS analysis was performed in a horizontal 11.7T magnet with bore size of 160 mm equipped with a gradient set capable of maximum gradient strength 750 mT/m and interfaced to a Bruker Avance III console (Bruker Biospin GmbH, Ettlingen, Germany). A volume coil (Bruker Biospin GmbH, Ettlingen, Germany) was used for transmission and a two‐element surface array coil for receiving (Rapid Biomedical GmbH, Rimpar, Germany). Isoflurane‐anesthetized mice (70% N_2_O and 30% O_2_; flow 300 mL/min, induction with 5%, maintenance 1.5%) were fixed to a custom‐made holder and positioned in the magnet bore in a standard orientation relative to gradient coils.

In order to detect the muscle volumes, high‐resolution anatomical images were acquired using a fat‐suppressed 3D gradient echo FLASH sequence with following parameters: TR = 25 msec, TE = 2.5 msec, flip angle 10°, matrix of 320 × 192 × 64, FOV of 26 × 20 × 36 mm^3^, and two transitions. Same acquisition was repeated at fat frequency, suppressing water resonance, to evaluate fat content from the combination of the two scans.

For determination of metabolite levels in muscle, a voxel of 1.5 × 2.0 × 2.5 mm^3^ was placed in the gluteus maximus (2.0 × 2.0 × 2.0 mm^3^ for gastrocnemius) of the mouse based on anatomical images collected as described above (Fig. 10A). Automatic FASTMAP shimming routine was used to adjust B0 homogeneity in the voxel. A PRESS sequence (TE = 10 msec) combined with outer volume suppression (OVS) was used for the prelocalization. Three OVS blocks were used interleaved with water suppression pulses. Data were collected by averaging 512 excitations (frequency corrected for each average) with TR of 2 sec, number of points 4096, and spectral width 5 kHz. In addition, a reference spectrum without water suppression was collected from the identical voxel using the same acquisition parameters. Peak areas for major resonances (Cr, Cho, Tau, and components for intra‐ and extramyocellular lipids) were analyzed using LCModel (Stephen Provencher, Inc., Oakville, Canada), and results are given relative to water content in tissue. MRI/MRS data were evaluated by an unpaired, nonparametric Mann–Whitney test comparing ranks using Graphpad Prism software.

### Open field

Exploratory activity in a novel environment was assessed by 1 h trials in a photocell‐equipped automated open field (41 cm × 41 cm × 30 cm; Versamax system, Accuscan Instruments). Measures were taken of total distance travelled and number of rearing movements. Activity chambers were contained inside sound‐attenuating boxes equipped with houselights and fans.

### Rotarod performance

Balance and motor coordination were evaluated on an accelerating rotarod (Ugo‐Basile, Stoelting Co., Wood Dale, IL). Revolutions per minute (rpm) were set at an initial value of 3, with a progressive increase to a maximum of 30 rpm across 5 min, the maximum trial length. The first test session consisted of three training trials, with 45 sec between each trial. Subsequent retests consisted of two trials, with a maximum time of 5 min per trial. Latency to fall, or to rotate off the top of the turning barrel, was measured by the rotarod timer.

### Wire hang test for grip strength

Each mouse was placed on a large metal cage lid. The lid was gently shaken to induce the mouse to grip the metal grid. The cage top was then inverted, and latency for the mouse to fall from the lid was recorded. The maximum trial length was 60 sec.

### Marble‐burying assay

Mice were tested in a Plexiglas cage located in a sound‐attenuating chamber with house light and fan. The cage contained corncob bedding 5 cm deep, with 20 black glass marbles (14 mm diameter) arranged in an equidistant 5 × 4 grid on top of the bedding. Animals were given access to the marbles for 30 min. Measures were taken of the number of buried marbles (designated as two third of the marble being covered by the bedding) by an observer blind to genotype.

### Water maze test

Mice were tested in the Morris water maze using the visible platform task. The water maze consisted of a large circular pool (diameter = 122 cm) partially filled with water (45 cm deep, 24–26°C), located in a room with numerous visual cues. Mice were first tested for their ability to find a visible escape platform (diameter = 12 cm) across 2 days of training. The criterion for learning was an average latency of 15 sec or less to locate the platform across a block of four consecutive trials per day. For each trial, the mouse was placed in the pool at 1 of 4 possible locations (randomly ordered), and then given 60 sec to find the cued platform. If the mouse found the platform, the trial ended, and the animal was allowed to remain 10 sec on the platform before the next trial began. If the platform was not found, the mouse was placed on the platform for 10 sec, and then given the next trial. Subsequent retests consisted of four trials, with a maximum time of 60 sec per trial. Measures were taken of latency to find the platform and swimming speed by an automated tracking system (Ethovision, Noldus Information Technology, Wageningen, the Netherlands).

In addition to the visible platform tests, mice were given a 5 min free swim at age 38 weeks, and a 6 min free swim at age 48 weeks, to determine swimming ability across a longer time period. The escape platform was removed from the pool during the free swim tests.

### Statistical analysis for standard motor assays

Data were analyzed using one‐way ANOVAs (analyses of variance) or repeated measures ANOVAs, with the factors genotype and either age or time (the repeated measures). Although the experimental groups included both male and female mice, subject numbers were not high enough to include sex as a separate factor in the behavioral analyses. However, because of expected overt group differences, males and females were analyzed separately for body mass. Group means were compared using post hoc Fisher's PLSD (protected least significant difference) tests only when a significant effect of genotype was found in the overall ANOVA. For all comparisons, significance was set at *P* < 0.05.

### Gait dynamics

Gait analysis was performed via ventral plane videography, as previously described in detail (Hampton et al. 2004; Kale et al. 2004; Maddatu et al. 2004; Hampton and Amende 2010) (Fig. 6). Briefly, we used a motor‐driven treadmill with a transparent treadmill belt (DigiGait Imaging System, Mouse Specifics, Inc., Quincy, MA). A high‐speed digital video camera was positioned below the transparent belt to focus on the ventral view of subjects walking on the belt. A polycarbonate compartment, ~7 cm wide × ~30 cm long (adjustable depending on the size of the animals and their speed of walking), was mounted on top of the treadmill to maintain the mice within the view of the camera. Images of the ventral surface of the mouse were digitally captured at a rate of ~150 frames per second. The color images were converted to their binary matrix equivalents and the areas of the approaching or retreating paws relative to the moving belt were calculated throughout each stride. The plotted area of each digital paw print (paw contact area) imaged sequentially in time provides a dynamic gait signal, representing the temporal record of paw placement relative to the treadmill belt. From each gait signal for each of the four limbs, numerous postural and kinematic metrics were compared. Each gait signal for each limb comprises a stride duration that includes the stance duration when the paw of a limb is in contact with the walking surface, plus the swing duration when the paw of the same limb is not in contact with the walking surface. Hind paw shared stance was computed as the mean of the durations during each hind limb recruitment that both hind limbs were in contact with the treadmill belt simultaneously. Paw placement angle was calculated as the angle that the long axis of a paw makes with the direction of motion of the animal during peak stance. Gait data were collected and pooled from both the left and right forelimbs, and the left and right hind limbs.

The treadmill speed was set at 24 cm/sec, and the orientation of the treadmill was horizontal. However, because neuromuscular deficits in mouse models can be subtle, we also challenged the animals with a more rigorous treadmill walking protocol in this study. The treadmill, walking compartment, and camera system were all pitched at an angle so that the animals walked up an incline of 15° (the angle of treadmill incline was based on published protocols) (Brussee et al. 1997; Whitehead et al. 2007) and the walking speed was set to 40 cm/sec. Approximately 5 sec of video were collected for each walking subject to provide more than 20 sequential strides. Each subject was allowed to explore the treadmill compartment for ~1 min with the motor speed set to zero. Only video segments in which the subjects walked with a regularity index of 100% were used for image analyses. The treadmill belt was cleaned between studies if necessary. DigiGait data are presented as mean *±* SE. Student's two‐tailed *t* tests were used to determine statistical differences between WT and dysferlin‐deficient mice. Experiment was carried out at 25 weeks of age. Gait differences were considered significant with *P *<* *0.05.

## Results

### Creatine kinase analysis in Bla/J mice

To characterize the Bla/J dysferlin‐deficient mouse model, 32 WT C57BL/6J mice were compared to 32 age‐matched Bla/J mice equally divided by gender unless otherwise noted. This large cohort was divided into two equal groups (*N* = 16/genotype) for independent analyses of circulating creatine kinase (CK) and locomotor function to assure the blood draws did not alter the physical examinations. Serum was analyzed for creatine kinase activity at 6, 9, and 12 months of age. As previously reported, at all time points tested, Bla/J mice had significantly higher serum creatine kinase than C57BL/6J controls, the increase of which ranged from three‐ to fivefold (Fig. 8). The increased serum CK activity in Bla/J mice was noted independent of gender.

### Traditional motor challenges in Bla/J mice

#### Open field

At 10 weeks of age, the control and Bla/J groups had comparable levels of locomotor activity in a 1‐hr open field test (Fig. 1A). However, when retested at the age of 15 weeks (Fig. 1B), the knockout mice showed significantly less locomotion, measured as total distance travelled (main effect of genotype, *F*(1, 28) = 10.24, *P* = 0.0034) (Fig. 1B). By the age of 57 weeks of age, the deficit in overall exploration of dysferlin‐deficient mice was more pronounced and significance was observed even at the earliest time point (main effect of genotype, *F*(1, 25) = 12.22, *P* = 0.0018) (Fig. 1C). By 66 weeks, the Bla/J mice were hypoactive at every time point across the 1 h period (post hoc tests following repeated measures ANOVA, main effect of genotype, *F*(1, 24) = 31.17, *P* < 0.0001) (Fig. 1D).

During the open field test, the number of times the mice stood on their hind legs with front paws raised (termed rearing) was quantitated over 60 min. The Bla/J mice demonstrated deficits in rearing during the activity tests (Fig. 2), with significant differences already detectable at age 10 weeks (genotype × time interaction, *F*(11, 308) = 2.05, *P* = 0.0242), and more pronounced at later time points during the second test at week 15 (main effect of genotype, *F*(1, 28) = 7.4, *P* = 0.0111; genotype × time interaction, *F*(11, 308) = 2.94, *P* = 0.001). Despite only mild differences in rearing between the control and knockout groups at 57 weeks (genotype × time interaction, *F*(11, 275) = 2.33, *P* = 0.0093) (Fig. 2C), at 66 weeks, the Bla/J mice had marked deficits in rearing at every time point in the session (main effect of genotype, *F*(1, 24) = 48.36, *P* < 0.0001; genotype × time interaction, *F*(11, 264)=4.53, *P* < 0.0001) (Fig. 2D).

#### Rotarod

Both the C57BL/6J and Bla/J mice showed initial high levels of proficiency on the accelerating rotarod, with no motor impairment found up to the age of 57 weeks (Fig. 3). At 66 and 71 weeks, Bla/J mice had significantly reduced latencies to fall, indicating the emergence of motor coordination deficits in dysferlin‐deficient mice (post hoc tests following repeated measures ANOVA, main effect of genotype, *F*(1, 24) = 8.12, *P* = 0.0088; genotype × age interaction, *F*(7, 168) = 3.59, *P* = 0.0013).

#### Wire hang test and marble‐burying assay

Similar to the findings from the rotarod task, the C57BL/6J and Bla/J mice had comparable levels of performance in tests for grip strength and marble burying during the first several months of the study (Fig. 4A and B). However, at 67 and 72 weeks, dysferlin‐deficient mice exhibited shorter latencies to fall in the wire hang test (post hoc tests following repeated measures ANOVA, main effect of genotype, *F*(1, 24) = 9.82, *P* = 0.0045; genotype × age interaction, *F*(5, 120) = 7.03, *P* < 0.0001) and a reduced number of total marbles buried (post hoc tests following repeated measures ANOVA, main effect of genotype, *F*(1, 24) = 6.25, *P* = 0.0196).

#### Water maze

Both the Bla/J mice and the controls were able to swim directly to a visible platform throughout the study, indicating good visual and swimming ability (Fig. 4C). However, starting at 38 weeks of age, the dysferlin‐deficient Bla/J group showed lower swim speeds than the C57BL/6J mice (Fig. 4D). The deficits in swim velocity were most overt at ages 67 and 72 (post hoc tests following repeated measures ANOVA, main effect of genotype, *F*(1, 22) = 12.56, *P* = 0.0018; genotype × age interaction, *F*(6, 132) = 12.48, *P* < 0.0001).

#### Body mass

The male C57BL/6J and Bla/J mice had comparable body mass from 9 to 72 weeks of age (Fig. 5). In contrast, the female Bla/J mice had significantly higher body mass than female controls at almost every age (post hoc tests following repeated measures ANOVA, main effect of genotype, *F*(1, 13) = 7.78, *P* = 0.0153; genotype × age interaction, *F*(15, 195) = 4.05, *P* < 0.0001) (Fig. 5B). Despite similar body mass noted for the male Bla/J mice, analyses of the wet mass of individual muscles indicated that, specifically, the psoas *P* ≤ 0.0001 and the gluteus *P* = 0.0002, displayed the most significant difference, being smaller in dysferlin‐deficient male mice (Fig. 5C). Quadricep muscles of Bla/J mice were also significantly smaller than C57B6 mice *P* = 0.0211. Sixteen Bla/J male mice and 16 C57B6 male mice were evaluated to obtain these data.

### DigiGait analysis of Bla/J mice

In addition to the traditional physical tasks described above, a separate cohort of Bla/J mice were compared to WT at 8 months of age using the DigiGait observation chamber which collects more than 30 metrics of gait and posture kinematics during horizontal and graded treadmill activity (*N* = 6 per genotype). The fore and hind paw placement angles and the hind paw shared stance were compared between BLa/J and WT mice. The data demonstrate significant differences in all of these parameters depending on the incline of the running platform (Fig. 6). In addition, running at a slower speed on a horizontal plane showed marked differences in placement angle of both fore and hind paws between the Bla/J and WT cohorts. At a faster speed, the placement angles were similar between genotypes. The hind limb shared stance angle was also observed to be significantly different in Bla/J mice running horizontal while not on a sloped platform.

### MRI/MRS of Bla/J mice

Muscle volumes were longitudinally evaluated using magnetic resonance imaging (MRI) in Bla/J and C57BL/6J mice. Figure 9A shows representative images at the same anatomical level of a single female Bla/J mouse at 6, 9, and 12 months of age. At 9 and 12 months of age, gross abnormalities were visible in MR images from mouse hip muscles including volumetric reduction of gluteus and psoas and fat infiltration (Fig. 9A and B). In water images (Fig. 8A, gray scale images, 6–12 months) black band (yellow arrows) are assigned as fat confirmed by fat frequency scan (water–fat fuse image, red color). No significant loss of muscle volume was detectable in gastrocnemius or tibialis anterior of Bla/J compared to control mice throughout the time course of the study (Fig. 9B). Cellular metabolism was also measured in gluteus and gastrocnemius muscles using MRS. Aligned representative MRS scans showed increasing content of extramyocellular lipid (EMCL) 1.5 ppm in Bla/J gluteus muscle (Fig. 10A and B), but not in the gastrocnemius (Fig. 10C). EMCL 1.1 ppm was also significantly increased in the gluteus at all time points evaluated, but intramyocellular lipid levels were similar in BLA/J and C57BL/6J mice at 6 and 9 months of age (Fig. 11). At 12 months of age in BLA/J gluteal muscles, the fatty acid content overwhelmed the spectrum, and IMCL data could not be specifically defined. Muscle metabolites (creatine, cholines, and taurine) were also evaluated in these time points in BLA/J and C57BL/6 gluteus maximus, normalized signal intensity for creatine and cholines were significantly lower in Bla/J mice at the 6‐month time point and nonsignificantly different thereafter; taurine on the other hand was significantly lower in Bla/J mice at both 6‐ and 9‐month time points, unfortunately the fatty acid spectrum prevented Bla/J mice metabolites from being defined at 12 months of age.

## Discussion

The major findings in this study were the discovery of early locomotor deficits in Bla/J mice which could allow for faster treatment screenings (Table 1). In particular, gluteal muscle wasting was found to be the most salient, measurable indicator of muscle deterioration in Bla/J mice. Further exploration of the gluteus muscles could lend insight into the selective phenotype also seen in humans (Petersen et al. 2015). Current dysferlin‐deficient mouse models, including the Bla/J mouse, fail to provide a complete representation of human dysferlinopathy. Disease onset in humans normally occurs during adolescence; CK levels in humans are at least 20‐fold higher than normal levels, and at approximately 20 years of age, patients are generally nonambulant. In the A/J, SJL/J, and Bla/J dysferlin‐deficient mice models, serum CK activity is also elevated, yet, to a much lesser extent (three‐ to sixfold; Fig. 8) (Ueyama et al. 2002; Ho et al. 2004). However, variability in CK levels in SJL/J mice was also reported making this assay in SJL/J mice less reliable for dysferlinopathy studies (Rayavarapu et al. 2010). Regarding locomotor activities, to date, there has been no dysferlin mouse model that mimics the nonambulant human condition. Furthermore, dysferlin‐deficient mouse models also have accompanying pathologies not associated with human dysferlinopathy, such as aggressive behavior and increased risk for lymphoma in SJL mice, while AJ mice possess more severe abdominal muscle wasting phenotype (Weller et al. 1997; Ho et al. 2004). Taken together, these variable and subtle disease phenotypes complicate the evaluations of potential therapeutics by largely focusing on histological correction which may or may not be associated with disease progression in humans. In this work, a comprehensive long‐term evaluation of locomotor activity in a single dysferlin‐deficient model was undertaken to define consistent locomotor deficits.

For assessing behavioral deficits related to dysferlinopathy, tests herein included extended periods of exploration, rearing, rotarod, grip strength, swimming speed, marbles buried, and were performed once a month to track activity, endurance, strength, and coordination. Although significant differences were apparent at older ages, in general, prolonged motor challenges under elective conditions resulted in the greatest locomotor deficits using these standard assays. In particular, tracking of free exploration and rearing activity for an hour demonstrated significant differences between 15‐week‐old Bla/J and WT mice after the initial 30 min (Figs. 1 and 2). This difference in male mice could not be simply attributed to increased body mass; however, female dysferlin‐deficient mice demonstrate a modest body mass increase throughout these experiments (Fig. 5A). The gluteus and psoas degenerate phenotype, due to their importance for both rearing and open field distance propulsion, could also help account for these assays being significant at an earlier age.

Longitudinal MRI experiments revealed progressive muscle deterioration restricted to specific hip muscles; psoas and gluteus muscles visibly atrophy from 9 months onward, whereas changes in tibialis anterior and gastrocnemius regions were not observed (Fig. 9A). It is worth noting that above statement is an observation of muscle atrophy outlining the muscle borders, but there exists another apparent ongoing process that seems to be specific to gluteus; muscle tissue wasting and fat replacement that starts *inside* the muscle (Fig. 9A). These images are acquired using fat‐suppressed 3D gradient echo sequence, which reveals a band of absent normal muscle tissue replaced with fat (Fig. 9, black appearing holes, yellow arrows). This is confirmed by repeating the acquisition with same sequence but with modification of suppressing the water frequency and measuring from the fat frequency (Fig 9A, colocalized water–fat fused image). Furthermore, in our MR spectroscopic experiments, the initiation of fat infiltration in gluteal muscles can be observed as early as, and prior structural visibility in MR images, 6 months of age using EMCL 1.5 ppm resonance as a reporter (Fig. 10B). Despite this fat accumulation, the wet muscle mass, specifically the gluteal and psoas muscles, were significantly decreased compared to other tested distal muscles confirming that Bla/J mice have limb‐girdle muscular dystrophy (LGMD).

An investigation of 10 LGMD2B patients showed that patients gait abnormalities started to emerge 7 years after disease onset (Mahjneh et al. 2001). The patients' lower limbs became externally rotated with the development of weakness in the hips, and the upper limbs become intrarotated after a period of 10 years after disease onset. Similarly, we observed a wider angle of rotation for both the hind paw and fore paw of the Bla/J mice compared to the C57BL/6 using the DigiGait (Fig. 6). Walking at high speeds, uphill or downhill, also amplified the degree of rotation of the paws and a slight decrease in the stride length of Bla/J males was observed. LGMD2B patients do not experience alterations in stride length early in the disease, but stride width is affected due to weakness in the proximal muscles of the lower limbs (Mahjneh et al. 2001). This suggests that the muscle involvement observed in mice is relevant to human observed muscle deterioration.

Despite the absence of an animal model that identically mimics human dysferlinopathy, the existing lines offer particular advantages and disadvantages in the development in therapeutics. Disease onset in A/J or Bla/J lines manifests later than the SJL/J models, but this allows for the study of histological and behavioral studies before disease onset. In developing therapeutics, the SJL/J model would make it hard to determine how much recovery of muscle function from therapeutics is possible, due to the pathological onset in infancy. The severity of SJL/J model would also make it difficult to determine the toxicity of therapeutics. Furthermore, it is possible that accompanying immune deficiencies observed in the SJL/J mice contribute to disease progression, rather than the loss of dysferlin itself (Bernard and Carnegie 1975). Therefore, with a more severe disease progression than the AJ model, and lacking additional phenotypic abnormalities present in the SLJ mouse, the Bla/J mouse is the most suitable model to investigate the effectiveness of therapeutics, before or after disease onset. This would be particularly attractive when using rearing and open field testing, as it can detect differences as early as 15 weeks in Bla/J mice, making it possible to screen therapeutics without long study times.

While the Bla/J mouse model has the potential to save time experimentally, the model may be of particular relevance with similar age of symptom onset as human dysferlinopathy. Humans begin showing symptoms in early adulthood, which is consistent with the Bla/J mouse model showing phenotypic differences starting around 15 weeks of age. Studies have showed there is a disabled lipid and glucose uptake/metabolism in Bla/J similar to primary human patient dysferlinopathy myoblasts, suggesting possibly a shared mechanistic phenotype (Keller 2014). This is particularly interesting as human metabolism markedly changes after puberty from oxidative to glycolytic predominance (Taylor et al. 1997; Timmons et al. 2003, 2007; Stephens et al. 2006; Armstrong and Barker 2009). Evaluating lipid abnormalities, we quantified intramyocellular lipids (IMCL) and extramyocellular lipids (EMCL). While IMCL showed no significant difference between groups, we found EMCL were significantly increased only in Bla/J muscles that underwent muscle wasting such as gluteal and psoas muscles. Previous literature has shown lipid infiltration is a feature also seen in human dysferlinopathy, yet has not been reported in other muscular dystrophies such as calpainopathy, DMD, and myotonic dystrophy (Grounds et al. 2014). Further investigation of the relevance of this correlation, as well as pathway elucidation, is still necessary to demonstrate whether lipid infiltration could be future therapeutic target for dysferlinopathy.

The propensity for sarcoma development has been reported in *mdx* ^*−/−*^ and *Dysf* ^*−/−*^ mice, and is postulated to result from DNA damage caused by overactive muscle regeneration (Schmidt et al. 2011). In fact, A/J mice have a predisposition to rhabdomyosarcomas, while SJL/J mice have an increased prevalence of lymphoma (Weller et al. 1997; Sher et al. 2011). Consistent with these previous reports, we observed a predisposition of proximal hind leg masses in Bla/J mice that appear to be tumors upon histological analysis compared to none observed in the C57BL6 controls (DNS) (Schmidt et al. 2011; Sher et al. 2011; Hosur et al. 2012).

In summary, this work presents a comprehensive longitudinal study of muscle size and function in Bla/J mice. This model of LGMD shows progressive loss of muscle strength, especially during prolonged activities, and interestingly, significant deterioration of the GM as evidenced by MR imaging and spectroscopy. Although the fat accumulation underlines the gluteal muscle wasting phenomenon, it is worth noting that the volume reduction of gluteal and psoas muscle volumes provides one of the most significant, although not earliest, windows for the model characterization using noninvasive imaging. The consistent motor/phenotypical deficits defined herein may allow noninvasive monitoring of dysferlinopathy progression following the administration of potential therapeutics while lending further insight into the disease condition.

## Conflict of Interest

T. H. is owner of Mouse Specifics, Inc., a purveyor of instrumentation to study gait analysis.
